# Successful Phakic Intraocular Lens Implantation with the Usage of Topical Ascorbic Acid in Patient with Reduced Corneal Endothelial Cell Density

**DOI:** 10.3390/medicina58101367

**Published:** 2022-09-28

**Authors:** Hung-Chi Chen, Chia-Yi Lee, Chao-Min Cheng, Yi-Jen Hsueh, Chao-Kai Chang, Wei-Chi Wu

**Affiliations:** 1 Department of Ophthalmology, Chang Gung Memorial Hospital, Linkou 333423, Taiwan; 2 Department of Medicine, Chang Gung University College of Medicine, Taoyuan 33302, Taiwan; 3 Center for Tissue Engineering, Chang Gung Memorial Hospital, Linkou 333423, Taiwan; 4 Institute of Medicine, Chung Shan Medical University, Taichung 40201, Taiwan; 5 Nobel Eye Institute, Taipei 100008, Taiwan; 6 Department of Ophthalmology, Jen-Ai Hospital Dali Branch, Taichung 41265, Taiwan; 7 Institute of Biomedical Engineering, National Tsing Hua University, Hsinchu 300044, Taiwan; 8 Department of Optometry, Yuanpei University of Medical Technology, Hsinchu 30015, Taiwan; 9 Department of Optometry, Da-Yeh University, Chunghua 51500, Taiwan

**Keywords:** ascorbic acid, endothelial cell density, corneal endothelium, phakic intraocular lens

## Abstract

We aimed to describe the use of topical ascorbic acid (AA) in a patient with reduced endothelial cells density (ECD) who was scheduled for phakic intraocular lens (pIOL) implantation. A 28-year-old woman presenting with dry eye and reduced ECD would like to have her high myopia (spherical equivalence >−15.0 D) corrected. The procedure of laser refractive surgery or even pIOL was not indicated for the reduced ECD of 1865/mm^2^ in the right eye and 2188/mm^2^ in the left eye, as well as level 3 dry eye. Fortunately, the ECD increased to 3144/mm^2^ in the right eye and 2538/mm^2^ in the left eye after topical AA was prescribed for one year preoperatively and one month postoperatively, with concomitant improvement of dry eye to level 1. Finally, bilateral pIOL implantation was performed smoothly and no sign of corneal decompensation was found postoperatively. Three months postoperatively, the ECD showed a satisfactory level of 2983/mm^2^ in the right eye and 3003/mm^2^ in the left eye. In conclusion, topical AA instillation might increase and maintain the density of central human corneal endothelial cells (HCECs) even after pIOL implantation.

## 1. Introduction

Phakic intraocular lens (pIOL) implantation has been applied to correct high myopia for decades, particularly with better refractive outcomes than laser refractive surgery such as laser in situ keratomileusis for high refractive errors greater than 8 diopters (D) [1]. On the other hand, either iris-fixated pIOL or iris-claw pIOL might lead to corneal endothelial damage and even corneal decompensation, in which case the pIOL might need to be removed [2,3,4]. Although endothelial keratoplasty can be used to manage corneal decompensation [5], chances of postoperative cataract formation and graft dislocation limit the visual quality for those phakic individuals undergoing anterior chamber pIOL implantation [5,6].

Ascorbic acid (AA) used to be utilized to treat corneal alkali burns without significant side effects [7,8] and also has the ability to scavenge free radicals from phacoemulsification and prevent corneal endothelial damage in animal models [9]. Recently, perioperative application of topical AA has been shown to prevent injury in human corneal endothelial cells (HCECs) and corneal decompensation in patients with endothelial cell density (ECD) lower than 1500/mm^2^ and pre-existing HCEC disorders including Fuchs endothelial corneal dystrophy and cytomegalovirus endotheliitis [10]. In addition, AA has been demonstrated to induce HCEC proliferation and prolong the lifespan of HCECs significantly in vitro [11] and to enhance the level of ECD postoperatively [12]. For some reason, patients with reduced ECD would undergo intraocular surgery which may compromise the corneal endothelium. Whether AA has protective or even proliferative effects for such clinical scenarios needs to be verified.

Here, we report a 28-year-old woman with reduced ECD, but who still underwent pIOL implantation without following corneal decompensation, probably due to perioperative topical AA with increment of central ECD. Informed consent with reference to examples of potential severe complications such as bullous keratopathy, persistent ocular hypertension, uveitis and retinal detachment was signed by the patient after thorough discussion.

## 2. Case Presentation

**Figure 1:**
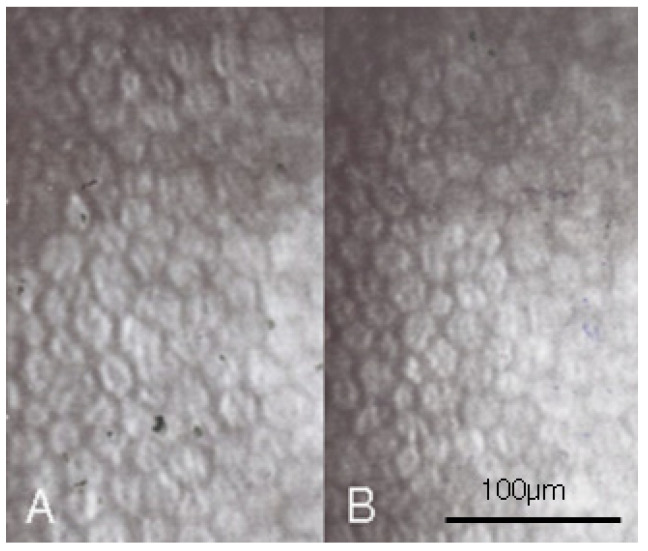
The initial endothelial cell morphology one year preoperatively with nominal magnification of 400×. (**A**) The endothelial morphology of right eye. (**B**) The endothelial morphology of left eye.

**Figure 2:**
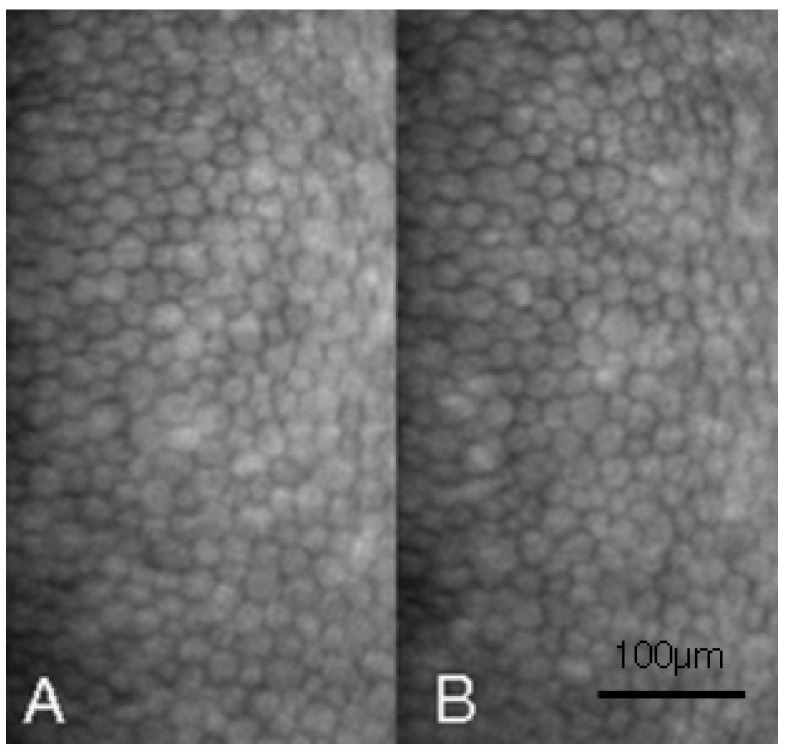
The endothelial cell morphology after topical ascorbic acid instillation immediately before surgery with nominal magnification of 400×. (**A**) The endothelial morphology of right eye. (**B**) The endothelial morphology of left eye.

A 28-year-old woman with high myopia presented with bilateral blurred vision since childhood and recently developed bilateral halo, with a strong desired to have the refractive error corrected. On examination, the best-corrected visual acuity (BCVA) was 20/25 in the right eye and 20/20 in the left eye and the intraocular pressure measured by pneumatic tonometry (Topcon c60, Topcon Corp, Tokyo, Japan) was 15.5 mmHg in the right eye and 15.1 mmHg in the left eye. We measured the intraocular pressure three times and calculated the average value. The manifest refraction after trial lens showed high myopia with cycloplegic spherical equivalent of −15.75 D in the right eye and −16.75 D in the left eye and the autorefractor (Nikon NRK 8000, Inc., Tokyo, Japan) revealed cycloplegic spherical equivalent of −16.50 D in both eyes. In addition, diffuse corneal punctate lesions was observed via silt-lamp bio-microscopy in both eyes and a non-contact in vivo specular microscope (CEM-530, Nidek, Gamagori, Japan) demonstrated a coefficient of variation of 52 in the right eye and 40 in the left eye, hexagonality of 33% in the right eye and 69% in the left eye and ECD of 1865/mm^2^ in the right eye (Figure 1A) and 2188/mm^2^ in the left eye (Figure 1B); thus, bilateral dry eye (level 3) and reduced ECD were diagnosed. To show more details of the specular microscope exam, we used the same device for all pictures., taken by one experienced technician, and one image was captured in each exam, but the technician would take an additional image if the primary picture was not clear enough to yield the four HCEC parameters. The technician has been working in this field for more than 10 years and is the leader of the optics department in our hospital. The technician measured the corneal endothelium with focus on the central 3 mm of cornea, and the obtained images were transmitted into the software program provided by the manufacturer which automatically counted approximately 200 cells for each analysis to yield the ECD value. After thorough discussion with the patient, laser in situ keratomileusis was not safe due to high myopia and pIOL implantation was also risky due to the reduced ECD. Routine lubricants and topical AA diluted in balance salt solution (50 mg/mL, QID) were then administered for one year to maintain the AA concentration for an adequate period. No adverse effect was found during the whole interval. The specular microscopy illustrated coefficient of variation of 42 in the right eye and 46 in the left eye, hexagonality of 46% in the right eye and 63% in the left eye, and the ECD resumed to a satisfactory level of 3144/mm^2^ (Figure 2A) in the right eye and 2538/mm^2^ in the left eye (Figure 2B). Intraocular pressure of 15.6 mmHg in the right eye and 16.1 mmHg in the left eye was observed.

**Figure 3:**
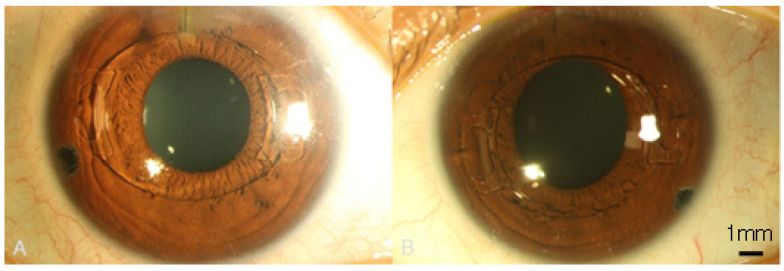
The clear cornea appearance with phakic intraocular lenses one week postoperatively. (**A**) The external eye image of right eye. (**B**) The external eye image of left eye.

**Figure 4:**
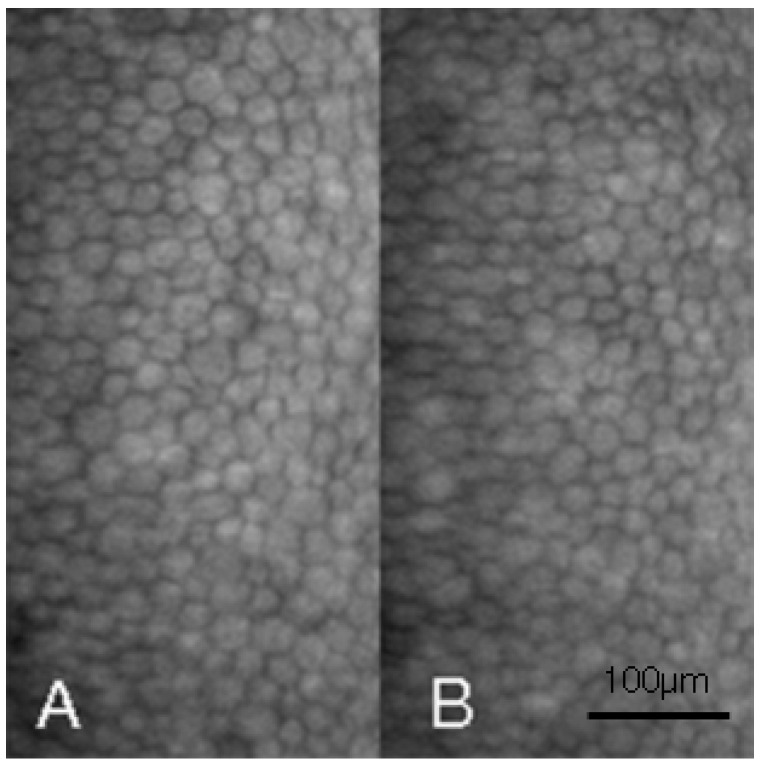
The endothelial cell morphology three months postoperatively with nominal magnification of 400×. (**A**) The endothelial morphology of right eye. (**B**) The endothelial morphology of left eye.

**Figure 5:**
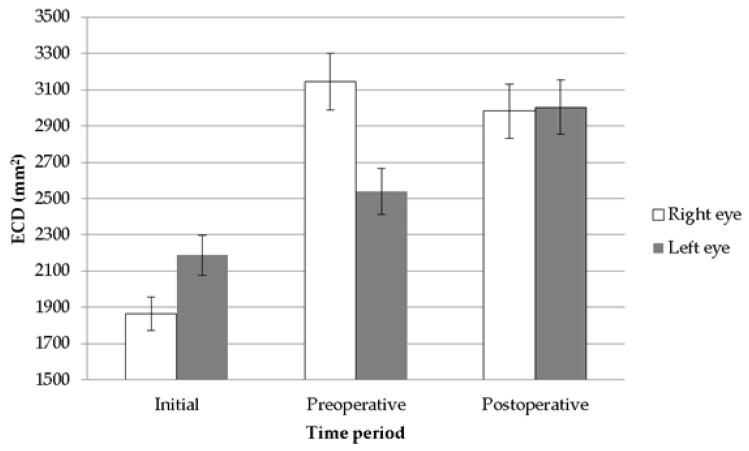
The bar chart of the endothelial cell density change throughout the study period.

Accordingly, bilateral pIOL implantation was arranged. The axial lengths were measured with an optical biometry (IOL master 500, Zeiss, Oberkochen, Germany) and showed 29.32 mm in the right eye and 29.37 mm in the left eye. We used Barrett Universal II and SLK/T formula to calculate the IOL power and both keratometric values from autorefractor and topography (CA-800, Topcon Corp, Tokyo, Japan) were considered in the formula. Bilateral implantation of PMMA IOLs (Artisan aphakia model 205, OPHTEC, Schweitzerlaan, NR Groningen, The Netherlands) with body length of 5.4 mm and overall length of 8.5 mm was carried out smoothly. Postoperatively, both eyes showed no signs of corneal edema, but pIOL in situ one week postoperatively (Figure 3A,B). Surprisingly, spherical equivalent showed an almost emmetropic value of 0.13 D in the right eye and −0.13 D in the left eye with BCVA of 20/20 in both eyes. Topical AA instillation was kept for one month postoperatively. In the last visit at three months postoperatively, BCVA stayed at 20/20 in both eyes, while the specular microscopy showed a coefficient of variation of 37 in the right eye and 36 in the left eye, hexagonality of 59% in the right eye and 63% in the left eye, and ECD remained at a fairly high value of 2983/mm^2^ in the right eye (Figure 4A) and 3003/mm^2^ in the left eye (Figure 4B). The intraocular pressure was 13.4 mmHg in the right eye and 14.2 mmHg in the left eye. The trends of ECD changes in both eyes are shown in Figure 5.

## 3. Discussion

The application of AA to manage various diseases originates from the fact that it has anti-oxidative ability and it is an ideal ocular nutritional supplement [13,14]. In systemic diseases, intravenous AA has proven as an effective and safety management agent in the retardation of severe sepsis and the extent of multiple organ failure [15]. In addition, AA can also be applied to patients with cancer to reduce the symptoms including fatigue, pain and mood disorders to improve the quality of life [16]. Concerning ophthalmic disorders, AA has been used for age-related macular degeneration, cataract and diabetic macular edema [17,18,19]. For the cornea, AA showed effectiveness in treating alkali burn injury and preserving the corneal stroma as well as the endothelium in ocular procedures [7,8,20,21]. In a two-case report, perioperative instillation of topical AA up to one month effectively prevented endothelial injury resulting from phacoemulsification-induced oxidative stress in patients with reduced ECD due to Fuch’s corneal endothelial dystrophy and corneal endotheliitis [10]. Unfortunately, the fellow eye of case 1 in this research did not receive preoperative topical AA and bullous keratopathy did develop, necessitating subsequent endothelial keratoplasty [10]. In addition to the anti-apoptotic activity of AA in HCECs [11], AA may also have the potential to trigger the growth of HCECs, which is supported by the current case report.

**Figure 6:**
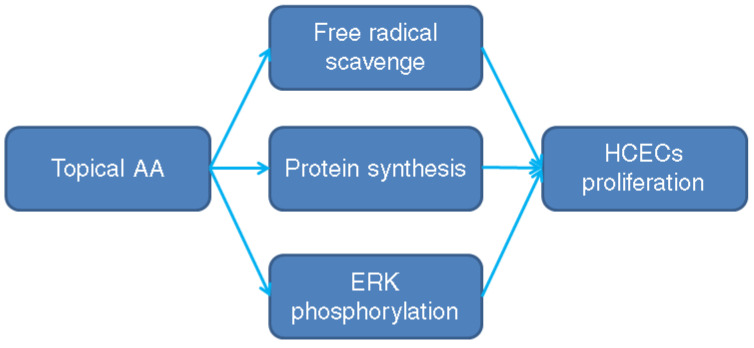
The possible mechanism of ascorbic acid on human corneal endothelial cell proliferation. AA: ascorbic acid, HCEC: human corneal endothelial cells, ERK: extracellular signal-regulated kinases.

While AA induces proliferation of HCECs in culture medium according to previous studies [11,22], other antioxidants such as retinyl acetate, glutathione and sodium alpha-tocopherol phosphate, do not exhibit such effects [22]. In an in vitro study by Shima et al., the AA enhanced HCEC proliferation and the confluence of HCECs was achieved in about 2–3 weeks, with an increment in HCEC lifespan [11]. According to our recent publications, AA application led to proliferation of corneal endothelial cells via GLUT1-ERK pathway in a rabbit model [23], and the proliferation of HCECs after AA instillation was observed clinically, with the ECD two-fold compared to that measured before AA treatment [12]. In the current report, an increment of HCECs with higher ECD was found in both eyes one year after the topical AA treatment. Moreover, ECD did not decrease prominently after the pIOL implantation while the ECD in the left eye was even higher postoperatively. We stopped applying AA to the patient one month after the surgery and the ECD remained above the average for healthy individuals in the Chinese population [24]. To our knowledge, this is the preliminary evidence that AA increases the number of HCECs clinically, which still persisted after the pIOL implantation. The reason for the proliferative effect of AA on HCECs is unknown, possibly due to free radical scavenge, certain protein synthesis or extracellular signal-regulated kinases (ERK) phosphorylation in the rabbit corneal endothelium [23] (Figure 6). The AA concentration we administrated was 50 mg/mL which was higher than the AA concentration in regular culture medium. However, the HCECS is immersed in aqueous humor and he bioavailability of topical eyedrops is only 5%. Accordingly, we utilized a much higher AA concentration four times a day to achieve similar AA concentration in culture medium. This concentration of AA mimics the antioxidant effect in culture media and protects the HCECs during and after the pIOL implantation. The pH of our topical AA may be an issue but no ocular surface damage was found in our experimental study using the rabbit (unpublished data). Still, only one image for each measurement in our patient is inadequate and the fact that a non-contact specular microscope can only measure the central ECD are two major limitations of our study. Our concept that AA could lead to ECD increment needs further study for verification.

The pIOL implantation can correct myopic refractive error up to −20 D and astigmatism less than 2.5 D [25], which is more suitable for our patient than laser refractive surgery [1]. Still, pIOL implantation may result in corneal decompensation in patients with fair ECD above 2500/mm^2^ [4]. In our patient, the level of ECD was only about 60 to 70% that of ordinary healthy individuals in the Chinese population [24]. Although the ECD was not very low compared to that of those undergoing phacoemulsification in previous reports [10,26], there was still a chance to develop corneal decompensation after the intraocular procedure. As a result, topical AA was still applied to our patient for one month postoperatively to ensure the normal level of ECD.

In conclusion, the perioperative instillation of topical AA might increase the density of central HCECs in patients with reduced ECD, which might remain stable even after pIOL implantation.

## Data Availability

The data in this study is not available since the image may invade the privacy of our patient.
